# USING NATURALLY OCCURRING QUALITATIVE DATA IN GERONTOLOGICAL CANCER RESEARCH

**DOI:** 10.1093/geroni/igac059.591

**Published:** 2022-12-20

**Authors:** Sean Halpin, Michael Konomos

**Affiliations:** Evidera, Decatur, Georgia, United States; Emory University, Atlanta, Georgia, United States

## Abstract

Cancer therapies for older adults have accelerated at breakneck speed in the last few decades, necessitating evaluation of their delivery and uptake to ensure patients receive their maximum benefit. Among the vast array of evaluation tools available, those utilizing naturally occurring data—data produced without intervention from a researcher—are a powerful but underused tool. In this presentation, we will review two methods for examining naturally occurring data, participant observation and conversation analysis (CA), in an educational intervention study of multiple myeloma patients receiving autologous stem cell transplant. First, we will review how participant observation of nurse-led education visits (n=70) was incorporated to iteratively improve video-based education. Next, we will review use of CA in reviewing audio recordings containing reference to the education videos of 12 nurse-led education visits (1011 minutes of audio). Ultimately, understanding the purposes of and ways of using naturally occurring data have potential for improving the evaluation of patient education.

